# Triple Burden in the Democratic Republic of Congo: Climate Change, Armed Conflict, and the Silent Spread of Arboviruses. A Narrative Review

**DOI:** 10.1002/hsr2.72188

**Published:** 2026-03-29

**Authors:** Stanis Donnang, Hermann Yokolo, Dujardin Makeda, Joshua Ekouo, Zubayer Shams, Johan Armel Domga Kaptso, Josias Silatchom Kamgang, Yann Valerin Waffo, Christian Tague

**Affiliations:** ^1^ Université Libre Des Pays Des Grands Lacs Goma DR Congo; ^2^ Inclusive Development Research Center (IDRC) Goma DR Congo; ^3^ Department of Research Medical Research Circle (MedReC) Goma DR Congo; ^4^ Brunel Medical School Brunel University London UK

**Keywords:** arbovirus, armed conflict, climate change, epidemic, public health

## Abstract

**Background:**

The Democratic Republic of Congo (DRC) faces a “triple burden” of public health threats which includes climate change, protracted armed conflict, and the under‐recognized spread of arboviral diseases. Arboviruses, such as dengue, chikungunya, Zika, and yellow fever, are transmitted by *Aedes aegypti* and *Aedes albopictus* mosquitoes. Certain factors, like environmental changes, population displacement, and inadequate vector control, have created conditions that favor sustained transmission.

**Methods:**

The narrative review was conducted to compile evidence on factors influencing arbovirus spread in the DRC and to identify the priority causes of prevention. A comprehensive literature search was done in PubMed and Google Scholar for studies, NGO reports, and government documents published between 2019 and 2025 in French or English, focusing on human populations in the DRC or similar contexts.

**Results:**

Key findings demonstrate that yellow fever remains endemic, with recurrent outbreaks and a case fatality rate of up to 21.3% in past years. Seroprevalence studies show significant dengue exposure (up to 41% in Kinshasa), multiple chikungunya epidemics which affected millions, and intermittent Zika virus exposure. On the other hand, adverse climate change alters vector ecology and increases transmission potential through drought, floods, rising temperatures, and deforestation. Armed conflict proved to play a major role in the large‐scale displacement of people into overcrowded, unsanitary environments. This, as a result, disrupts the surveillance and control programmes and increases vulnerability to arboviruses.

**Conclusion:**

The convergence of climate change, armed conflict, and arbovirus transmission poses a growing public health threat in the DRC. Strengthening integrated and reinforced surveillance, augmenting diagnostic capacity, applying sustainable vector control, and advocating climate and security considerations in health policy are essential and mandatory. Without coordinated and multidisciplinary action, the silent and hazardous spread of arboviruses will remain consistent to weaken and compromise health system resilience.

## Summary

- The Democratic Republic of Congo (DRC) confronts a *triple health crisis* facilitated by adverse climate change, ongoing armed conflict, and emerging arboviral infections.
- Climate change in the DRC is speeding up vector proliferation and extending transmission seasons, which in turn expands arbovirus‐endemic zones.
- Ongoing armed conflict causes disruption in healthcare delivery and displacement of populations, increasing extreme vulnerability to infectious disease outbreaks.
- Arboviruses such as dengue, Zika, yellow fever, and chikungunya are often underdiagnosed due to limited laboratory capacity and overlap of symptomatic manifestations with malaria.
- In addition, climate‐driven ecological and epidemiological shifts establish ideal conditions for silent arbovirus spread and contribute to risk for the human population.
- These problems can be eradicated by coordinated surveillance, aggressive vector control, and humanitarian‐health integration, which are essential to break the cycle of vulnerability and unsafe situations.

## 1 Introduction

**Figure 1:**
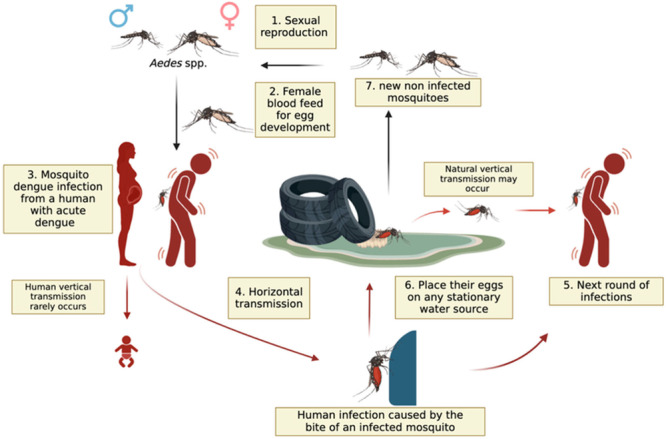
Reproduction cycle of *Aedes* spp. and dengue transmission.

Today, arboviruses are a real public health problem for many countries in sub‐Saharan Africa, including the Democratic Republic of Congo. These arboviruses are transmitted by different families of mosquitoes: the Aedes mosquito, including the species *Aedes aegypti* and *Aedes albopictus*. These mosquitoes generally bite during the day and lay their eggs in stagnant water (Figure 1). These vectors transmit dengue virus, Zika virus, chikungunya virus, and yellow fever virus [1, 2]. In recent decades, arboviral diseases have experienced a global resurgence, largely due to factors such as climate change, population displacement to risk areas, and the limitations of vector control systems [2, 3]. According to the WHO, mosquito‐borne viral infections, including arboviruses, have become a real global public health problem in recent years, particularly in tropical and subtropical countries where ecological conditions favor the proliferation of vectors [4, 5]. Factors such as climate change, armed conflicts, and the environmental crisis could exacerbate the spread of arboviruses in the DRC, highlighting the need for strengthening prevention and health management measures in this region [4, 6]. This article aims to highlight the impact of climate change and armed conflicts on the rapid spread of arboviruses in the DRC and propose the necessary courses of action for their prevention.

## 2 Methodology

This narrative review was conducted with the aim of synthesizing the available knowledge on the different factors favoring the spread of arboviruses and proposing perspectives and means of control. A literature search was conducted in electronic databases such as PubMed and Google Scholar. The search period covered publications from 2019 to 2025, in French and English. Our inclusion criteria were articles on human populations, climate change, armed conflicts and arboviruses in the DRC or similar contexts; articles published between 2019 and 2025; academic publications, NGO reports, and government documents.

### 2.1 Search Strategy and Study Selection

A structured narrative search strategy was applied. The databases PubMed and Google Scholar were searched using combinations of the following keywords: “arbovirus”, “dengue”, “chikungunya”, “Zika”, “yellow fever”, “Aedes”, “climate change”, “armed conflict”, “displacement”, and “Democratic Republic of Congo”. Boolean operators (“AND”, “OR”) were used.

The search covered publications from January 2019 to January 2025 in English or French.

Inclusion criteria were: (i) studies or reports addressing arboviral diseases in human populations; (ii) studies conducted in the DRC or comparable sub‐Saharan African settings; (iii) peer‐reviewed articles, governmental documents, or reports from international or non‐governmental organizations.

Exclusion criteria included animal‐only studies, conference abstracts without full text, and publications lacking primary epidemiological or contextual data.

A total of 214 records were identified, of which 137 remained after removal of duplicates. Following title and abstract screening, 62 records were assessed in full text, and 33 sources were included in the final synthesis.

## 3 Epidemiology of Arboviruses in the Democratic Republic of Congo

### 3.1 Yellow Fever

Yellow fever remains a major public health concern in the Democratic Republic of Congo (DRC), due to its vector‐borne transmission and epidemic potential. According to data from the DRC National Committee, 75 cases of yellow fever, including 16 deaths (a case fatality rate of 21.3%), were recorded in the country until September 1, 2016 [7, 8]. This situation has prompted an intensification of surveillance and response efforts. More recently, the World Health Organization (WHO) reported a total of 1577 suspected cases of yellow fever recorded between 2021 and 2024, with 55 deaths reported, corresponding to an estimated case fatality rate of 3.5% [9]. In 2024, between the first and 48th epidemiological week, the DRC reported 452 cases, of which 18 were laboratory‐confirmed and 434 were considered suspect (Table 1). Data sources include WHO weekly epidemiological records, bulletins, and published epidemiological studies. No deaths were associated with the cases reported during this period [10, 11]. These data illustrate not only the persistence of virus circulation within the country, but also the importance of epidemiological surveillance and preventive vaccination measures to limit the impact of this preventable disease.

**Table 1:** Epidemiology of the main arboviruses in the DRC.

Arbovirosis	Main epidemiological data	Years	Specific remarks
Yellow fever	75 cases (16 deaths, lethality 21.3%), 1577 suspected cases (55 deaths, lethality 3.5%), 452 cases (18 confirmed, 434 suspected, 0 deaths)	2016, 2021–2024, 2024 (Weeks 1– 48)	Endemic, importance of vaccination and surveillance
Dengue	68.8% seroprevalence (1960). Prevalence 2.7% to 8.8% (1998–2015), 41% (Kinshasa) and 23% (Matadi) in 2024, annual transmission 2.9% and 1.4%	1960, 1998–2015, 2024	Silent endemic circulation, poor clinical recognition
Zika	0% (RT‐PCR) on 453 sera (2003–2011), 3.5% (ELISA) on 978 samples (2013–2014), 1 confirmed case (PRNT)	2003–2011, 2013–2014	Low traffic, insufficient surveillance
Chikungunya	1st case in 1958, 2nd in 1960. 28.8% HIV positive in Kisangani (1998), 3 epidemics in Kinshasa (1999–2000): ~50,000 cases. 2023: 51 million suspected cases, 28,402 deaths	1958, 1960, 1998, 1999–2000, 2011, 2023	Massive circulation, major epidemic in 2023, underestimated

### 3.2 Dengue

Dengue fever now represents a major threat to global public health. According to the World Health Organization, nearly half of the world's population lives in at‐risk areas, with an estimated 100–400 million infections recorded each year [12, 13]. In the Democratic Republic of Congo, the first case of dengue fever was reported in 1960 in the territories of Ango and Bondo, located in the Haut‐Uélé province. At that time, a study revealed a high seroprevalence of 68.8% in the local population, reflecting intense viral circulation [5]. Between 1998 and 2015, four studies conducted in the country highlighted a serological prevalence of dengue variant between 2.7% and 8.8%, suggesting silent but persistent endemic circulation [5]. More recently, a study conducted in 2024 on a total of 3580 samples revealed a seroprevalence of IgG antibodies against the dengue virus of 41% in Kinshasa and 23% in Matadi, with annual transmission rates estimated at 2.9% and 1.4%, respectively [14]. These data suggest significant cumulative exposure in urban areas, despite low clinical recognition of the disease. This situation underlines the need for improved diagnostic capacities, epidemiological surveillance, and increased awareness within the Congolese health system to better understand the true extent of dengue in the country. Interpretation of seroprevalence data must be approached with caution due to well‐documented serological cross‐reactivity among flaviviruses, particularly between dengue, Zika, and yellow fever viruses. Enzyme‐linked immunosorbent assays (ELISA) may overestimate exposure in endemic settings where multiple flaviviruses co‐circulate. Neutralization assays such as PRNT remain the reference standard for specificity but are rarely available in routine surveillance in the DRC, limiting the precision of sero‐epidemiological estimates [14, 15].

### 3.3 Zika

In the Democratic Republic of Congo, data on Zika virus circulation remain limited, although a few studies have attempted to assess its presence in the population. Between 2003 and 2011, a first study was conducted on 453 serum samples collected across the country. None of these samples revealed the presence of Zika virus in RT‐PCR analyses, suggesting an absence or very low viral circulation during this period [5]. A second study, carried out between 2013 and 2014 on 978 blood samples collected in various regions of the DRC, revealed that 34 samples (3.5%) were positive for anti‐ZIKV antibodies using the ELISA test. However, only one of these 34 samples was confirmed positive by the plaque reduction neutralization test (PRNT), which is considered more specific [5]. These results suggest a one‐off and probably long‐standing exposure to the Zika virus, but they do not allow us to conclude that the virus is actively circulating in the country. This highlights the need to strengthen virological surveillance and diagnostic capacities to better understand the dynamics of this virus in the DRC.

### 3.4 Chikungunya

Chikungunya virus has been circulating for several decades in the Democratic Republic of Congo, although its importance has long been underestimated due to the lack of specific surveillance. The first two documented cases were reported during a concurrent yellow fever epidemic in the northeastern region of the country: the first in 1958 in Haut‐Uélé province, and the second in 1960 in Bondo territory, in the neighboring Bas‐Uélé province [5]. In 1998, during a West Nile virus epidemic in Kisangani, a serological study conducted on 45 patients revealed that 28.8% of them carried antibodies against Chikungunya virus, indicating probable viral co‐circulation [13]. Subsequently, the city of Kinshasa experienced three epidemic waves between 1999 and 2000, with approximately 50,000 suspected cases recorded during this period [5]. A new epidemic outbreak was confirmed in Matadi in 2011 using virological and serological analyses, which also highlighted previous circulation of the virus in the region [16]. More recently, in 2023, an outbreak of exceptional magnitude was reported, with more than 51,000 suspected cases of chikungunya reported nationwide, including 284 reported deaths, according to national surveillance summaries. The most affected provinces included Kinshasa, North Kivu, South Kivu, Ituri, Kasai, Kasai Central, Kwilu, Haut‐Katanga, Lomami, Kongo Central, and Sud‐Ubangi [16]. Furthermore, a serological study conducted between 2021 and 2022 in several municipalities of Kinshasa (Ndjili, Ngaliema, Mont Ngafula, and Limete) highlighted a serological prevalence of 15.3% for the dengue virus, confirming the active co‐circulation of arboviruses in the Congolese urban environment [16]. On the other hand, data on the Zika virus remain limited, largely due to the country's insufficient epidemiological and diagnostic surveillance capacities [17].

Other arboviruses have also been documented in the DRC. O'nyong‐nyong virus, an alphavirus transmitted by Anopheles mosquitoes, has historically caused outbreaks in Central and East Africa, including the DRC. In addition, Rift Valley fever virus circulation has been reported sporadically in neighboring countries, posing a potential spillover risk given livestock movement and ecological similarities. Data on these viruses in the DRC remain extremely limited, reflecting significant gaps in arboviral surveillance beyond the most recognized pathogens [18, 19].

## 4 Impact of Climate Change on the Spread of Arboviruses

Climate change is an aggravating factor in the dynamics of arbovirus transmission in Africa, particularly in the Democratic Republic of Congo. Entomological surveys have documented the expansion of Aedes aegypti and Aedes albopictus into peri‐urban and previously low‐risk regions, facilitated by urbanization, water storage practices, and environmental disruption. The increasing detection of these vectors in areas historically dominated by Anopheles species suggests a shift in vector ecology with direct implications for arbovirus transmission [20]. According to the World Health Organization, the global warming observed on the African continent slightly exceeds the global average, exposing African countries to more severe environmental, health, and social impacts [20].

In 2023, the DRC was ranked among the African countries most severely affected by drought [20]. This extreme situation increases the risk of the spread of arboviruses in affected regions. A 2018 study by Anna‐Bella Failloux demonstrated that drought can play a decisive role in the emergence of epidemics [21]. Indeed, it favors the migration of forest mosquitoes to urban areas in search of water [22]. Once in an urban environment, these mosquitoes, to survive dehydration conditions, must bite more frequently, thus increasing the risks of viral transmission. This phenomenon has been implicated in the yellow fever epidemic [21, 23]. Furthermore, rising temperatures directly influence the biology of vectors. It accelerates the life cycle of mosquitoes, particularly those of the genus *Aedes* (Figure 1), vectors of dengue, by increasing their reproduction rate and longevity [24, 25]. Furthermore, heat shortens the extrinsic incubation period of arboviruses inside the mosquito's body, which promotes faster transmission of pathogens to humans [21].

In addition to drought, extreme rainfall also contributes to the spread of arboviruses. Between November 2023 and January 2024, at least 18 provinces in the DRC were hit by heavy rains, resulting in significant flooding, particularly in Kinshasa, where more than 11,000 households were affected [25, 26]. These floods favored the formation of stagnant water, constituting breeding grounds for vector mosquitoes and thus facilitating the proliferation of arboviruses in urban and peri‐urban areas [27, 28]. Armed conflict has led to the destruction or closure of health facilities, interruption of laboratory supply chains, and loss of trained personnel in affected provinces. Surveillance activities are frequently suspended during periods of intense insecurity, resulting in delayed outbreak detection and under‐reporting of arboviral diseases.

Accelerated deforestation in the DRC, estimated at approximately 500,000 hectares of forest cover lost each year, is another worrying factor [29]. By profoundly modifying ecosystems, it disrupts the natural habitat of sylvatic vectors and pushes them closer to inhabited areas. This increased proximity between humans and vectors increases the risk of arbovirus transmission [20, 21]. Finally, climatic disasters such as floods often cause population displacements, favoring the geographical dispersal of vectors and facilitating the introduction of arboviruses into new regions where ecological conditions are favorable for their establishment [24, 25]. Thus, climate change appears to be a multifactorial catalyst for the spread of arboviruses in the DRC, making an integrated approach combining epidemiological surveillance, vector control, and environmental adaptation essential.

## 5 Influence of Armed Conflicts on the Spread of Arboviruses

Armed conflicts have historically played a significant role in the amplification of infectious diseases in the DRC, including arboviral diseases. A study by Guzmán et al. in 2003 on dengue fever and dengue hemorrhagic fever in the Americas highlighted that the post‐World War II epidemic of dengue hemorrhagic fever was linked to environmental degradation, mass population displacement, and waste from military equipment, which provided suitable habitats for mosquito vectors [30]. In the Democratic Republic of the Congo, prolonged armed conflicts, particularly in the eastern part of the country, have led to a major humanitarian crisis. Armed conflict in the DRC is not geographically uniform. While western provinces such as Kongo Central and parts of Kinshasa remain relatively stable, insecurity is predominantly concentrated in the eastern provinces, including North Kivu, South Kivu, Ituri, and parts of Tanganyika. These provinces account for the majority of internally displaced populations and experience the most severe disruptions to health services, surveillance systems, and vector control activities [30, 31, 32]. According to the World Health Organization, the escalation of violence has caused mass population displacement, a resurgence of infectious diseases, sexual violence, and considerable psychological distress [31, 32]. In this context of vulnerability, the spread of arboviruses has become a public health issue.

Population movements associated with conflicts promote the spread of arboviruses. Displaced persons often leave endemic areas, carrying vector mosquitoes, such as *Aedes aegypti*, to other regions previously less exposed [24, 33]. In addition, IDP and refugee camps are characterized by precarious living conditions: overcrowding, limited access to drinking water and sanitation, inadequate health care, inadequate temporary housing, and ongoing insecurity, particularly in terms of gender‐based violence [34, 35]. These conditions create an environment conducive to the transmission of arboviruses. Overcrowding in camps facilitates viral transmission when an infected mosquito can bite several individuals concentrated in the same confined space [33]. Furthermore, the lack of adequate drainage systems leads to the accumulation of stagnant water, which serves as breeding sites for vector mosquitoes [26]. The lack of prevention tools, such as insecticide‐treated mosquito nets or vector control programs, further increases the vulnerability of displaced populations [33]. It is also worth highlighting a factor specific to certain arboviruses, notably the Zika virus, which can be transmitted sexually [36]. In a context of armed conflict, where sexual violence is frequent, the risk of transmission of the virus by this route is increased. Many women and young girls, victims of rape in conflict zones, may thus involuntarily contribute to the spread of the virus within displaced communities [36]. In short, armed conflicts, by disrupting social organization and health systems, create fertile ground for the spread of arboviruses, highlighting the close interconnection between security instability, human vulnerability, and the emergence of infectious diseases.

Although mpox is not an arboviral disease, the 2022–2024 mpox public health emergency of international concern (PHEIC) centered in the DRC provides relevant operational lessons. These include delayed case detection due to limited diagnostic capacity, challenges in surveillance during population displacement, and difficulties in cross‐border coordination. These structural weaknesses are equally applicable to arboviral preparedness and response and illustrate the systemic vulnerabilities of the national surveillance architecture.

## 6 Perspectives and Recommendations

The resurgence of arboviruses in the Democratic Republic of Congo highlights the growing challenges facing the health system in a context marked by climate change, armed conflict, poverty, unplanned urbanization, and insufficient surveillance capacity. Faced with this complexity, multisectoral, integrated, and coordinated actions are essential.

1. Strengthening epidemiological and entomological surveillance: It is imperative to implement a robust national surveillance system capable of early detection of arboviral cases and monitoring the evolution of vectors. Expanding the network of laboratories capable of specific diagnosis (PCR, ELISA, PRNT) in the main risk areas is a priority.
2. Improving diagnostic and research capabilities: The DRC must invest in developing diagnostic infrastructure and train qualified personnel in virology, medical entomology, and epidemiology. Supporting local research will help us better understand the dynamics of arboviruses, their interaction with the environment, and their health impacts.
3. Integrated and sustainable vector control: The implementation of mosquito vector control programs must combine environmental sanitation, elimination of breeding sites, the use of insecticide‐treated nets, targeted insecticide spraying, and community awareness. An ecological and sustainable approach must be favored to avoid the emergence of resistance.
4. Integrating climate and security considerations into the health response: Prevention and response strategies must consider the effects of climate change (drought, floods) and displacement dynamics induced by armed conflict. This requires close collaboration between the health, environment, civil security, and humanitarian affairs sectors.
5. Strengthening community resilience: Awareness‐raising and health education campaigns must be strengthened to improve preventive behaviors, particularly in high‐risk areas and within displacement camps. Community engagement is essential for the success of prevention actions.
6. Advocacy and resource mobilization: The fight against arboviruses must be integrated into national health priorities and benefit from increased technical and financial support from international partners. Strong and transparent governance is necessary to ensure the sustainability of interventions.

## 7 Limitations of the Study

This review has some limitations, including the scarcity and heterogeneity of available data on arboviral diseases in the Democratic Republic of Congo, with uneven geographical coverage and variable study periods. Weak surveillance systems, particularly in rural or conflict‐affected areas, limit the representativeness of the data. The absence of recent analyses on emerging risk regions, as well as the lack of data on the socioeconomic and health impacts of arboviral diseases, are also notable limitations. Despite this, this review provides a useful synthesis to guide research and public health priorities.

## 8 Conclusion

The circulation of chikungunya, dengue fever, and, to a lesser extent, the Zika virus in the Democratic Republic of Congo reveals a worrying epidemiological reality, long underestimated due to weak surveillance systems and a lack of specific diagnostic tools. Available data show active and recurrent circulation of the chikungunya virus in several provinces, serological evidence of dengue transmission, and a lack of reliable information on the Zika virus. This situation highlights the urgent need to strengthen national capacities for detection, integrated surveillance of arboviral diseases, laboratory diagnostic confirmation, and the implementation of sustainable prevention strategies. In a context marked by climate change, rapid urbanization, increased human mobility, and health system failures, the DRC remains vulnerable to future arboviral epidemics. At an operational level, the DRC could prioritize the implementation of simplified differential diagnosis algorithms for acute febrile illness at the primary care level, integrating malaria, arboviruses, typhoid fever, and viral hemorrhagic fevers. Province‐level risk stratification based on climate vulnerability, vector presence, and displacement intensity would allow targeted deployment of diagnostics and entomological surveillance. Pragmatic upgrades, such as integrating arbovirus testing into existing GeneXpert or regional laboratory platforms, could provide rapid gains without requiring entirely new infrastructure. It is imperative to act at the scientific, institutional, and community levels to prevent and mitigate the impact of these neglected diseases on public health.

## Author Contributions

**Stanis Donnang:** conceptualization, methodology, investigation, and writing – original draft. **Hermann Yokolo:** data curation, formal analysis, writing – review and editing. **Dujardin Makeda:** software, validation, and visualization. **Joshua Ekouo:** investigation, resources, and data curation. **Zubayer Shams:** methodology, formal analysis, writing – review and editing. **Johan Armel Domga Kaptso:** writing – review and editing, investigation. **Josias Silatchom Kamgang:** validation and investigation. **Yann Valerin Waffo:** funding acquisition and resources. **Christian Tague:** conceptualization, supervision, writing – review and editing, final approval of the manuscript, supervision, and project administration.

## Funding

The authors have nothing to report.

## Conflicts of Interest

The authors declare no conflicts of interest.

## Transparency Statement

The corresponding author, Christian Tague, affirms that this manuscript is an honest, accurate, and transparent account of the study being reported; that no important aspects of the study have been omitted; and that any discrepancies from the study as planned (and, if relevant, registered) have been explained.

## Data Availability

The authors have nothing to report.
